# Occupational exposure to blood and body fluids and use of human immunodeficiency virus post-exposure prophylaxis amongst nurses in a Gauteng province hospital

**DOI:** 10.4102/hsag.v25i0.1252

**Published:** 2020-02-25

**Authors:** Melitah M. Rasweswe, Mmapheko D. Peu

**Affiliations:** 1 Department of Nursing Science, Faculty of Health Sciences, University of Pretoria, Pretoria, South Africa

**Keywords:** HIV PEP, occupational exposure, blood and body fluids, nurses, healthcare workers

## Abstract

**Background:**

Healthcare facilities in South Africa are confronted by several challenges arising from Human immunodeficiency virus (HIV) and acquired immune diseases syndrome infection pandemic. All categories of nurses continue to experience accidental occupational exposure to blood and body fluids (BBFs) of patients who are HIV-positive. Studies conducted revealed that nurses fail to report the occurrence of the exposures. This represents a serious challenge because they contract HIV infections whilst in the process of helping others.

**Objectives:**

The purpose of this study was to determine the occupational exposures and use of HIV post-exposure prophylaxis (PEP) amongst nurses at the selected tertiary academic hospital, Tshwane district, Gauteng province, South Africa.

**Methods:**

A quantitative descriptive study was conducted with 94 male and female clinical nurses, using a self-administered questionnaire that facilitated collection of biographical data, occupational exposures to BBFs and use of HIV PEP. The data analysis included univariate and bivariate descriptive analyses.

**Results:**

Of the 94 nurses, *n* = 40 (43%) had been exposed to BBFs, either through sharp or needle prick injuries or splashes but only 16 (46%) of them reported the incident. Nurses were not keen to report accidental occupational exposures to BBFs in their own facility and rather sought HIV PEP outside their workplace. They gave different reasons for their behaviour. For example, ‘I did not know where to report’.

**Conclusion:**

Our study highlights the gaps that exist in reporting occupational exposure to BBFs and obtaining HIV PEP. Therefore, we recommend evaluation of these occupational exposures to BBFs and the management thereof, as well as to address the identified problems.

## Introduction and background

Human immunodeficiency virus (HIV) is a deadly reality in South African healthcare facilities, where people living with HIV are receiving care. According to Meintjes et al. (2016), despite reduction in new infections, approximately 60% of patients admitted in South African hospitals are HIV-positive and 45% of them are receiving antiretroviral treatment (ART). Park (2016) indicates that large numbers of HIV-positive patients in the hospitals are because of increased availability of ART that transforms HIV and Acquired immune deficiency syndrome (AIDS) to chronic illness. This exposes the healthcare workers (HCWs) to the risk of contracting HIV through occupational health hazards whilst taking care of the infected patients. World Health Organization (WHO) guidelines (2013) reported that about 90% of the occupational exposures occur in Africa. Literature shows that nurses, especially those working in a high-prevalence HIV setting, are the most at risk of occupational health hazards (Agaba et al. 2012; Makhado & Davhana-Maselesele 2016). South African nurses, just like in rest of the world, are the major healthcare providers and bear the maximum load of providing care in the medical settings. They are thus at a greater risk of occupational exposure to blood and body fluids (BBFs) infected with blood-borne diseases such as HIV (Dhital, Sharma & Dhital 2017). This represents a serious concern as healthcare facilities in South Africa are confronted by several challenges arising from the HIV and AIDS infection pandemic, although the prevalence shows some variation by province or region (United Nations AIDS Special Analysis 2016).

To address this problem, WHO guidelines (2013) and South African National Department of Health (SANDoH) guidelines (2016) have presented universal precautions (UPs) that are strategies and interventions to protect against and prevent exposures to BBFs. The guidelines require all HCWs to use the available protection and treatment offered through HIV post-exposure prophylaxis (PEP) services if they are accidentally exposed to BBFs. Post-exposure prophylaxis measures are available to prevent HIV infection, which includes first aid, counselling, risk assessment, relevant blood samples investigations of the exposed person and source, as well as provision of short-term antiretroviral drugs within 2–72 h after accidental exposure for 28 days, along with follow-up evaluation (Moorhouse et al. 2015). The HIV PEP, if given within this timeframe, prevents the dissemination of virus in the body by inhibiting viral replication (Aminde et al. 2015; Bourry et al. 2010), reducing the risk of HIV infection. According to the WHO guidelines (2013), approximately 81% of the transmission of HIV risk can be reduced using PEP; however, the efficacy of PEP decreases with delayed initiation and is less effective if started after 72 h of exposure.

Human immunodeficiency virus PEP services are free and available in most of South African hospitals and clinics, so that HCWs can access the services immediately after accidental exposure to BBFs of the patients. However, several research studies show that many exposed HCWs fail to report the occurrence of the exposures (Ajibola et al. 2014; De Vries et al. 2011; Makhado & Davhana-Maselesele 2016; Mponela et al. 2015; Rossouw, Van Rooyen & Richters 2017; Sabermoghaddam et al. 2015; Sendo 2014). The researcher observed from the ongoing hospital statistics that the uptake of PEP was low, compared to accidental occupational exposures occurring in the wards.

## Aim of the study

The aim of the study was to determine the occupational exposures and use of HIV PEP amongst nurses working in highly specialised units such as intensive care unit (ICU), operating theatres (OTs), emergency department (ED) and maternity ward in a Gauteng province hospital, South Africa.

## Methods

### Design

The study design was a quantitative research approach using a descriptive survey.

### Study setting

The study was conducted in one of the public tertiary hospitals in Gauteng province, Tshwane district. The hospital has its own PEP service unit.

### Study population and sampling strategy

The study population was all clinical nurses (*n* = 312), consisting of day and night registered nurses, enrolled nurses and enrolled assistant nurses who were 18 years and older. The included nurses worked in ICU, OT, ED and maternity ward for at least 3 months during data collection. The study sample was selected using a systematic method. After obtaining a permission to conduct the study from the hospital management, the researcher requested a number of personnel working in casualty, maternity ward, ICU and OT. The researcher went to the selected disciplines to recruit the participants. Numbers were assigned against the names of those who shown interest to participate. Then, every third number or name on the list was circled until to the end of the list. All circled numbers or names were included in the study. To reach the required number of respondents, the same procedure was repeated on the remained number of nurses until the required respondents were reached. One hundred nurses were selected to participate in the study; however, only 94 nurses responded.

### Instrument

The questionnaire was developed by the researcher, supervisor and biostatistician after an extensive literature review on topic under study. The instrument was a structured questionnaire collecting information on biographical data characteristics and questions to determine nurse’s accidental occupational exposures to BBFs as well as use of HIV PEP services. The questionnaire was developed in English because all the respondents were fluent in English. The drafted questionnaire was submitted to the supervisors of the study at the university and the statistical consultant to examine and appraise all the component elements of the variables to ensure the relevance, appropriateness and adequacy of the questions. Problems identified were discussed, with corrections and changes made accordingly to improve the first draft. The final questionnaire was brief and clear, containing different types of open- and close-ended questions. A pretest was conducted amongst 10 clinical nurses who are not included in the main study, in order to assess the practicability of the instrument.

### Data collection

Data were collected using a questionnaire that was given to 100 selected nurses. All categories of nurses working in the casualty, maternity ward, ICU and OT of the selected hospital for 3 months and above were included. The researcher distributed the questionnaires to the nurses, who also signed informed consent form. The completion of questionnaire took between 30 and 45 min.

### Data analysis

The collected data were checked for completeness, and thereafter, it was captured using *Microsoft Excel* and converted into a *STATA 13* format for analysis. Descriptive statistics described and combined the collected data to simplify the task of interpreting and communicating the numerical information. Data were described and categorical variables were expressed as frequencies and percentages.

Univariate descriptive analysis, which included the frequency distribution of key items on the respondents’ accidental occupational exposures and use of HIV PEP services, was presented.

### Ethical considerations

Prior to the study, the researcher obtained approval and ethical clearance from the ethics committee of the Faculty of Health Sciences at the University of Pretoria (Ethical Clearance Number: 33/2014, 04/03/2014). Permission to conduct the study was granted by the management of the selected hospital. The study process considered the universal ethical principles of respect for persons, beneficence, non-maleficence and justice. Respondents were informed of the purpose, scope, benefits and risks of the study and their right to withdraw from participation if they wished. Nurses participated voluntarily and signed an informed consent prior to completion of a questionnaire. The questionnaire did not contain any information that can be traced back to the respondents, and completed questionnaires were posted anonymously in a sealed box placed at the corridors of the hospital. The name of the hospital was not divulged in any document, and the collected data were stored in a locked place to which only researchers and the biostatistician had access.

## Results

A total of 94 (*N* = 94) clinical nurses who were working in the ICU, OT, ED and maternity wards participated in this study. Male nurses were 22 (23%), whilst female nurses were 72 (77%). Sixty-six (70%) were registered nurses, 24 (26%) were enrolled nurses and four (4%) were enrolled assistant nurses. Most of the respondents, (52; 55%) possessed a diploma in nursing science. The biographical data are presented in Table 1.

**TABLE 1:** Biographical data of the respondents (*N* = 94).

Variable	Characteristic	Frequency	Percentage
Gender	Male	22	23
	Female	72	77
Total		94	100
Professional qualifications	Registered nurse	66	70
	Enrolled nurse	24	26
	Enrolled assistant nurse	4	4
Total		94	100
Level of education	Certificate	27	29
	Diploma	52	55
	Degree	12	13
	Honours	2	2
	Masters’	1	1
	PhD	0	0
Total		94	100

### Accidental occupational exposure and use of human immunodeficiency virus post-exposure prophylaxis

Out of the 94 respondents, 40 (43%) respondents had experienced occupational exposure to BBFs whilst performing their day-to-day work. The most exposed categories were registered nurses (32/66), enrolled nurses (7/24) and enrolled assistant nurses (1/4), respectively. The various procedures that result in nurses sustaining sharps injuries were found to be inserting intravenous infusion, administering injections, collision with one another whilst holding sharps or needles, cutting, disassembling or detaching instruments, disposing needles, recapping needles, assisting in theatre, drawing blood samples from patients or clients, waste disposal, delivering babies in clinical or emergency settings and suturing of wounds.

Out of 40 nurses who experienced accidental occupational exposure, only 16 (46%) nurses reported the accident to their supervisors in order to seek HIV PEP services, whilst 24 (54%) nurses did not report. All those who reported were assessed within 72 h and given the antiretroviral drugs for 28 days, but only seven completed the course of medication, and none of them finished the process of follow-up until discharged from HIV PEP unit.

**FIGURE 1:**
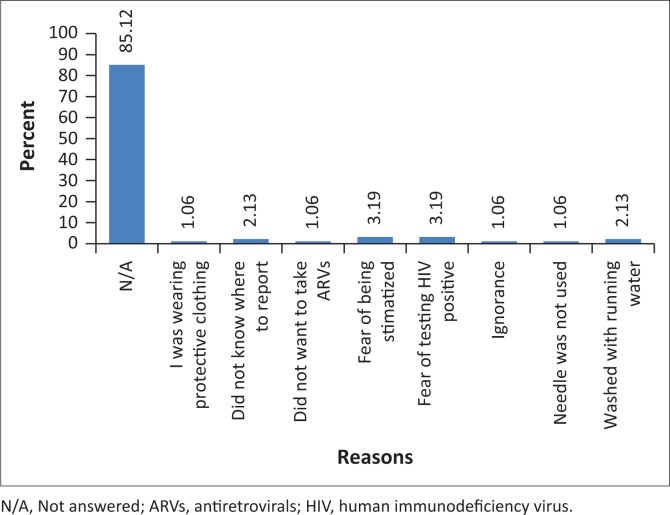
Reasons for not reporting incidents of occupational exposures (*N* = 94).

A follow-up question was asked to those who were accidentally exposed to BBFs and did not report the exposure to find out the reasons for not reporting. The results showed that about 80 (85.12%) of nurses did not answer this question, whilst 14 (14.88%) of those who responded gave different reasons for not reporting, such as ‘I did not know where to report’, ‘I was wearing protective clothing’, ‘I fear testing HIV positive’, ‘I fear to be stigmatised’, ‘I did not want to take ARVs’ ‘ignorance’, ‘needle was not yet used’ and ‘I washed the affected area with running water’. Out of 14 (14.88%) nurses who gave reasons for not reporting, three (3.19%) respondents feared to be stigmatised and three (3.19%) feared to test HIV positive as depicted in Figure 1.

**FIGURE 2:**
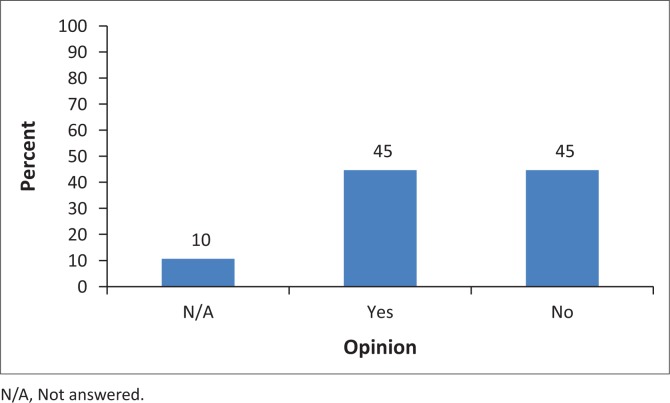
Respondents’ opinions as to whether nurses sought post-exposure prophylaxis services from other facilities or not (*N* = 84).

Nurses were further asked to give opinions on whether other nurses in their hospital could seek HIV PEP from their own facility or will rather go to other facilities for help. Figure 2 shows that 42 (45%) of the respondents agreed that other nurses sought PEP services from other facilities rather than in their own hospital and 42 (45%) respondents indicated that other nurses sought PEP services in their own facility, whilst 10 (10%) did not respond to the question.

## Discussion

This study has shown that occupational exposure to BBFs amongst nurses is frequent. Nearly half of the nurses reported exposures. The finding is comparable with the findings from other South African and international studies (Auta et al. 2017; Modupe et al. 2017; Nmadu, Sabito & Joshua 2016; Rossouw et al. 2017). In addition, a 2016 study on the knowledge and uptake of occupational post-exposure prophylaxis amongst nurses in Limpopo province demonstrated that occupational exposure to BBFs was common (Makhado & Davhana-Maselesele 2016).

The rate of notification of exposures was also low, as fewer respondents notified their exposure to the PEP services. In this study, 46% of study respondents reported the accident to sought PEP services. The results of this study are lower than the results of the study conducted amongst intern doctors in Gauteng province South Africa, whereby 83.5% of the intern doctors reported occupational exposures to sought out PEP services (Aigbodion, Motara & Laher 2019). The above findings are evidence that although occupational exposure to BBFs should be reported, there are still HCWs failing to report. Delay and failure to report exposures contribute to delay in initiating PEP and put HCWs and sexual partners at risk of infections transmitted through BBFs. Low reporting rates may cause underestimations of occupational exposures and provide wrong statistics. Underreported exposures will remain unknown leading to low rates of PEP uptake. Without reporting occupational exposures, PEP cannot be initiated. As Ndou (2017) also stated that this trend puts HCWs at risk of contracting infections whilst performing their daily work as the efficacy of prevention strategies cannot be analysed. The reasons for not reporting the exposures correlate with other studies conducted in Africa (Azadi, Anoosheh & Delpisheh 2011; Mathewos et al. 2013; Sendo 2014; Shriyan & Annamma 2012; Tabatabaei et al. 2016). Information on HIV PEP should be available at the unit level. Accessible information on HIV PEP services can improve the health of the HCWs and reduce disability and transmission of HIV.

Our study identified a significant gap between HIV PEP guidelines, implementation and practice. Despite the WHO and SANDoH guidelines, 54% of nurses working in the hospital were not following the guideline for reporting exposures. Other studies from the African continent have reported similar resistance to PEP despite national guidelines (Mbaisi et al. 2010; Owolabi et al. 2012). Many hospitals in developing countries are understaffed with high patient loads, similar to the hospital in our study. These conditions may contribute to high levels (43%) of occupational exposure as observed in this and other studies (Auta et al. 2017; Kassa et al. 2016; Ndou 2017).

Some of the nurses in this study preferred to seek HIV PEP from other facilities. The results are in line with the study of Papavarnavas et al. (2017), which revealed that some of the HCWs sought PEP services from other facilities to avoid being seen at their own facility PEP unit for confidentiality purpose. In addition, De Vries et al. (2011) observed that HCWs prefer to seek care far away from where they live or work because they need privacy and confidentiality. These results may imply that HCWs do not use their own hospital because the personnel working in the PEP unit are their peers and they doubt if confidentiality will be maintained. It is also clear that HIV and AIDS are still a stigma in South Africa (Haffejee, Ports & Mosavel 2016; Mohlabane et al. 2016). These can contribute to low reporting rate because of uncertainties of confidentiality amongst South African nurses and other HCWs.

### Limitations

Our study was conducted in a single hospital in Gauteng province of South Africa, and nurses from ICU, OT, ED and maternity ward were the only participants. Nurses in other wards of the same hospital and other hospitals around South Africa may give different results. Future studies should be conducted in other healthcare settings and hospitals and include a larger sample of other HCWs in Gauteng for generalisation. Our research was based on responses, and data could not be verified through other records such as the registry for occupational injury and PEP documents. Some of the respondents did not answer all the questions leading to a non-response bias.

### Recommendations

Nurses and other HCWs should be informed of the importance of knowing own HIV status and consider prevention of HIV infection as a personal responsibility regardless of HIV status. Human immunodeficiency virus–negative HCWs need to know PEP protocols. Human immunodeficiency virus–positive HCWs need to be encouraged to access in-hospital HIV services without fear of stigma, in order to improve their health status and reduce disability and transmission of HIV.

Healthcare workers should be encouraged to follow the HIV PEP guidelines to report all types of exposures to BBFs. The hospital management should ensure that there is adequate testing, proper management and follow-up of the affected. The Department of Health in collaboration with the hospital management should ensure adequate nursing staff establishment to roll out PEP services. There should also be periodic in-service training, education and awareness for all staff to be updated on issues related to infection prevention or current safety practices and occupational risk reduction. The institutional HIV PEP service should be accessible to all HCWs. The staff working in the PEP unit should improve on confidentiality to encourage reporting rate. The measures put in place to manage HCWs exposed to BBFs need to be evaluated and improved.

## Conclusion

Credible evidence in determining occupational exposure to BBFs and uptake of PEP services is important. Evidence can help to design and implement strategies that will increase the reporting and usage of HIV PEP services to prevent occupational transmission of HIV infection. The findings of this study serve to enhance the current understanding of the occupational exposures and practices of HIV PEP in the selected hospital. Our study also highlights the gaps that exist in reporting occupational exposure to BBFs and implications for the effective management of HCWs who are exposed to BBFs that may contain other infectious diseases. The poor utilisation of PEP services in the selected hospital suggests further inquiry regarding HIV PEP.
